# Three-tier quick-response code: Applications for encoded text and counterfeit prevention system

**DOI:** 10.1016/j.mex.2024.102585

**Published:** 2024-01-29

**Authors:** Sara Ignacio-Cerrato, David Pacios, José Miguel Ezquerro Rodriguez, José Luis Vázquez-Poletti, Nikolaos Schetakis, Konstantinos Stavrakakis, Alessio Di Iorio, María Estefanía Avilés Mariño

**Affiliations:** a Optics Department, Faculty of Optics and Optometry, Universidad Complutense de Madrid, Calle Arcos de Jalón, 118 28037, Madrid, Spain; b Department of Computer Architecture and Automation, Faculty of Informatics, Universidad Complutense de Madrid, Calle del Prof. José García Santesmases, 9, Madrid 28040, Spain; c Quantum Innovation Pc, Chania 73100, Greece; d Alma Sistemi Srl, Guidonia, Rome 00012, Italy; e Departamento de Lingüística Aplicada a la Ciencia y a la Tecnología, Universidad Politécnica de Madrid, Calle de José Gutiérrez Abascal, 2, Madrid 28006, Spain; f Department of Production Engineering and Management, Technical University of Crete, Kounoupidianna, Chania 73100, Greece

**Keywords:** Coding, Color, RGB, Multiplexed, Color Quick Responses Codes coding

## Abstract

This paper introduces a novel approach for encoding information in PDF documents or similar files. The proposed encoding involves a dual-step method: firstly, the information is encoded in base64, and subsequently, it is uploaded in a user-selected color, while the rest of the colors contain dummy information. Merging of the encoded segments results in a single QR code. The Literature Review subsection investigates the usage of similar methods for information encoding, followed by a comparison of the luminance of the generated QR code with theoretical expectations. Finally, diverse use cases are presented.

The proposed methodology is presented:

• Compare the results obtained from the theorical approximation with those acquired in the merged QR code.
• Use cases: encoding text sample to obtain a counterfeit system.
• Results, contributions, and future work.

## Graphical abstract

This figure represents the functionality of the application and the process expressed in the methodology. It has been divided the app part and the code functions in order to explain step by step all the codification process.

**Figure:**
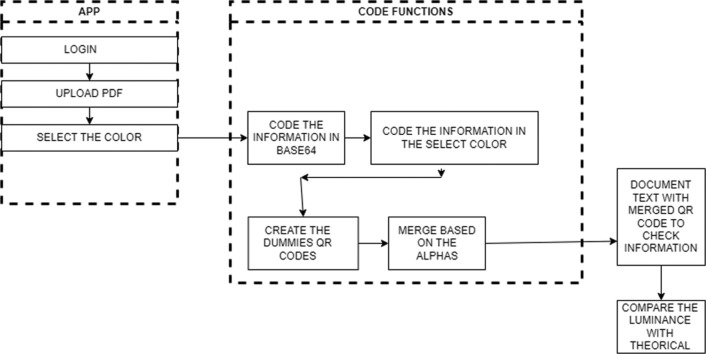

Specifications table

**Table:** 

Subject area:	Computer Science
More specific subject area:	*Codification*
Name of your method:	*Color Quick Responses Codes coding*
Name and reference of original method:	S. I. Cerrato, D. Pacios, J. M. E. Rodríguez, J. L. Vázquez-Poletti, N. Schetakis, K. Stavrakakis, A. Di Iorio, and M. E. A. Mariño, ``Secure and Efficient Transmission of Spatial Data using Colored Quick Response (QR) Codes: A Case Study for the EyE Project,'' in Frontiers in Optics + Laser Science 2023 (FiO, LS), Technical Digest Series (Optica Publishing Group, 2023), paper FD6.6.
Resource availability:	Software: https://figshare.com/s/da910ba721cbca686770

## Method details

### Literature review

Coding is the main part of the security to save the information from others. In this part we will introduce the literature which we have search to give us an idea what implies our methodology.

In terms of using QR Codes as coding system we have found several articles related with it. But the most of them there are related with two levels of codification [1,2,3] meanwhile our approach it's a third level system.

The first mentioned article uses the XOR approach [1] to save the information in a two level QR Code. It's a very interesting approach because they change the structure of the QR Code to save more information. The second mentioned article changes the structure of the QR code patterns to save more amount of information. They change the black patterns with a linear pattern [2] to code the information. The third mentioned article uses a polynomial [3] codification to encrypt the information in a two-dimensional QR Code.

This study presents a methodology to code information based on color, compared the luminance of the QR obtained with one theorical extracted from OpenCV [4,5,6] and different uses cases which can be used. The main idea is to code the information in three different QR Codes. The user will choose which color has the main information and the other will have dummy information. This process of the codification will be done twice, first code all the information in base64 and to save this information in a certain QR code.

## Methodology

**Fig. 1:**
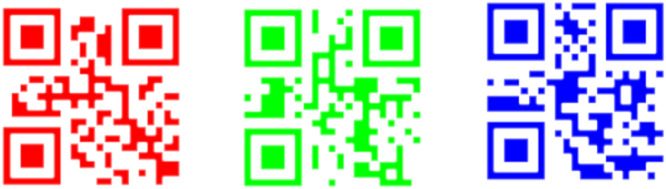
Generated QR codes: Red, green and blue.

This methodology uses the image combination provided by OpenCV [4,5,6] to create a coding system based on QR codes. The QR Code merged has the information provided in the three QR codes. This is the main key to code the information. On the other hand, we could identify any differences and analyze the luminance between the two separate performances by juxtaposing the theoretical and QR Code obtained merged the three QR codes. First, we build the QR codes [7] shown in Fig. 1 using the RGB coordinates from Eqs. (1), (2), and (3).

(1) $$\bar{R}=\left[255,0,0\right]$$

(2) $$\bar{G}=\left[0,255,0\right]$$

(3) $$\bar{B}=\left[0,0,255\right]$$

Then, we apply the formula provided by the Documentation to obtain the theoretical color values from the QR codes.

(4) $$\bar{C}=\alpha_{r}\;x\;\bar{R}+\;\alpha_{g}\;x\;\bar{G}\;+\;\alpha_{b}x\;\bar{B}$$

Being α determined by us to mix a certain percentage of each color. This α controls the chromaticity in each channel and it cannot be greater than 1. In this case the α are the following:

(5) $$\alpha_{r}=0.4$$

(6) $$\alpha_{g}=0.3$$

(7) $$\alpha_{b}=0.3$$

### Calculation the luminance of the sample: the influence of α. in the chromaticity

The phrase usually refers to the α. channel [8] in images, which denotes a pixel's luminosity. A value of 0 indicates that the pixel has minimum luminosity, whereas a value of 1 (in an 8-bit per channel image) indicates that it has maximum luminosity [9,10,11]. The following equation can calculate this pixel luminosity:

(8) $$L^{i,j}=\frac{\left(\alpha_{r}^{i,j}\times R\right)+\left(\alpha_{g}^{i,j}\times G\right)+\left(\alpha_{b}^{i,j}\times B\right)}{3}$$

Where *i* and *j* are the pixel coordinates, *L* is the total luminance and *R, G* and *B* are the vectors corresponding to the chromatic coordinates.

This total luminance can vary depending on the contribution of each channel. For example, if all channels participate, the distribution follows Eqs. (1), (2) and (3) with the values of the α. shown in the Eqs. (5), (6) and (7). For a particular case where all 3 channels contribute, it could be calculated as follows:

(9) $$L^{i,j}=\frac{\left(0.4\;\times \;255\right)+\left(0.3\;\times \;255\right)+\left(0.3\;\times \;255\right)}{3}=85$$

**Fig. 2:**
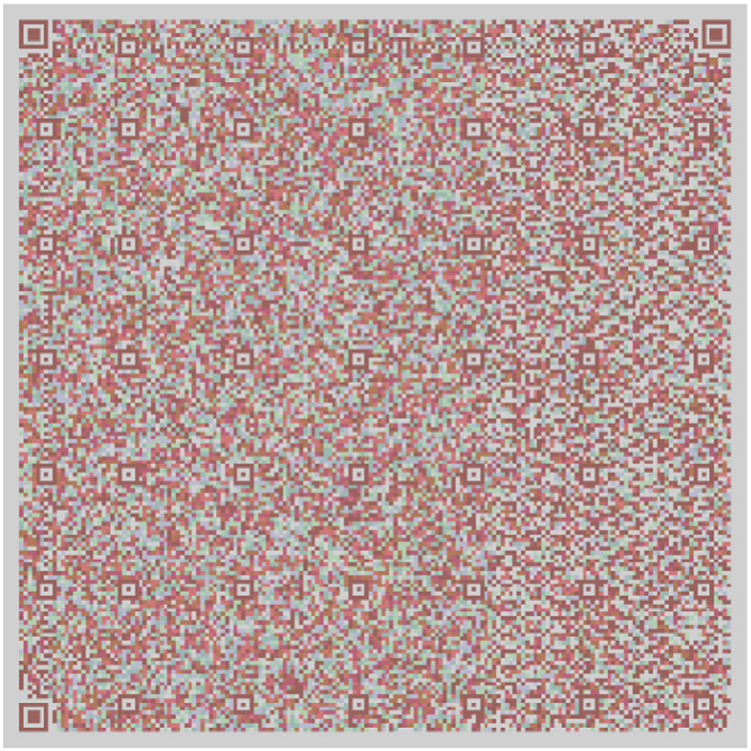
QR code obtained from different vaes of α..

When images are blended or superimposed, the alpha channel substantially impacts on chromaticity. Fig. 2 shows the influence of this alpha in the luminance of the image.

The value of alpha is critical because it controls the luminance of the sample and determines the color of the mixing.

## Use case

### Coding text sample

The previously mentioned ideas of encoding and decoding QR codes will be used in this use case to demonstrate how these techniques can inject information into QR codes of various colors and then decode them to recover the original information. To encode the report, a chapter from Miguel de Cervantes *Don Quijote de la Mancha* will be taken, and the functions mentioned above will be used to divide the text into information blocks (each QR codes monochromatic has a limit of 1200 characters), encode each block into a QR code [12,13,14] of a specific color, and then join the QR codes. In total, this color codification could store 3600 characters.

**Fig. 4:**
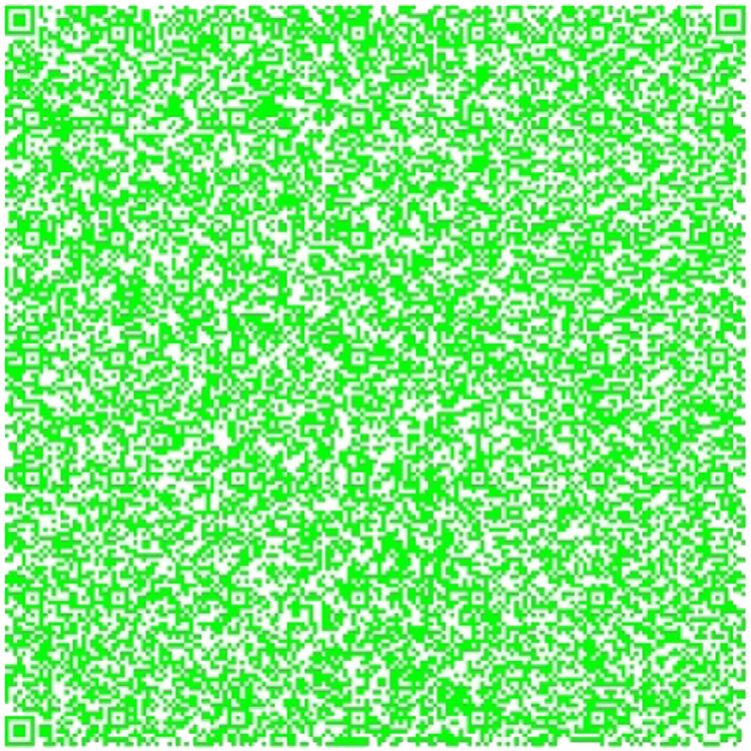
Part 2 of Chapter One of El Quijote Book.

**Fig. 5:**
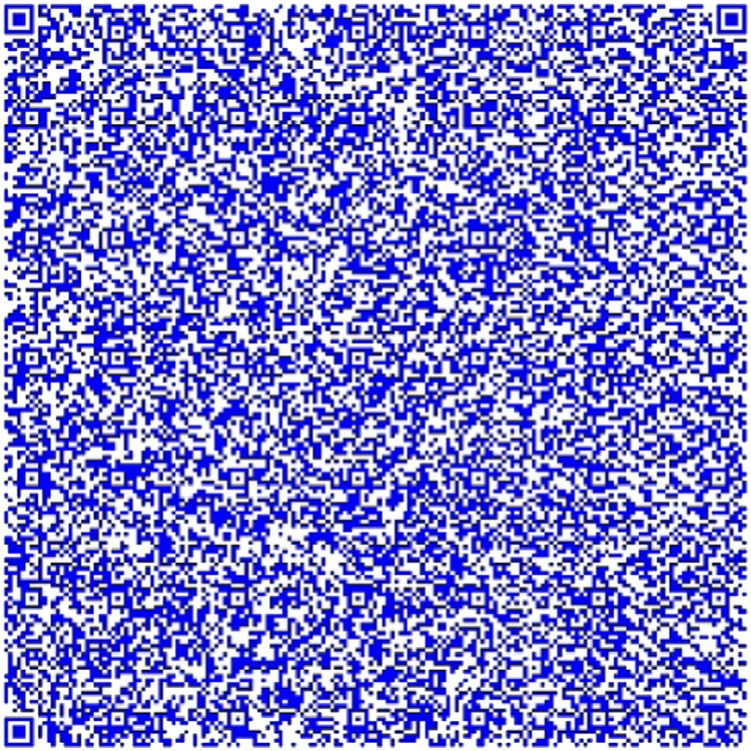
Part 3 of the Chapter One of El Quijote Book.

**Fig. 6:**
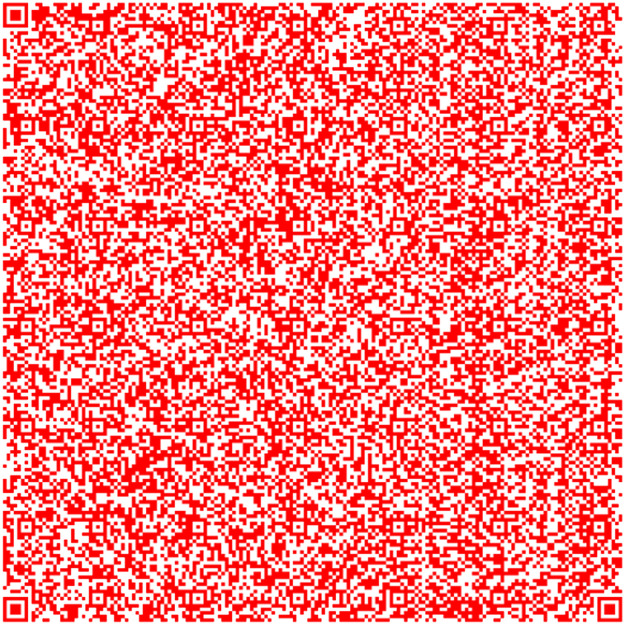
Part 1 of Chapter One of El Quijote Book encoded in base64.

The QR in Fig. 4 has information taken from the Quijote, the QR [15,16,17] in Fig. 5 contains information different from the other, and the QR in Fig. 6 contains information extracted from the text.

Once the full image of the various colored QR codes has been generated, they can be decoded to recover the original information from the Quixote chapter. This will show how QR codes can be used to encode information effectively and how decoding techniques can be used to get this information.

Once the complete image of the different colored QR codes has been generated, they can be decoded using the techniques above mentioned to retrieve the original information from the

Quijote chapter. This will demonstrate how QR codes can be used to effectively encode information and how decoding techniques can be used to retrieve this information.

### Anticounterfeiting application

**Fig. 7:**
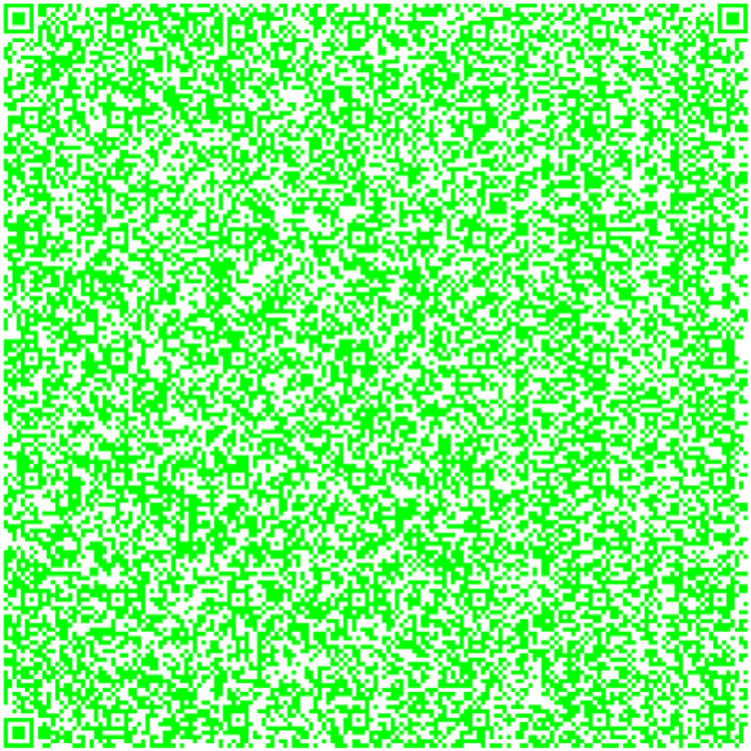
Part 2 of Chapter One of El Quijote Book encoded in base64.

**Fig. 8:**
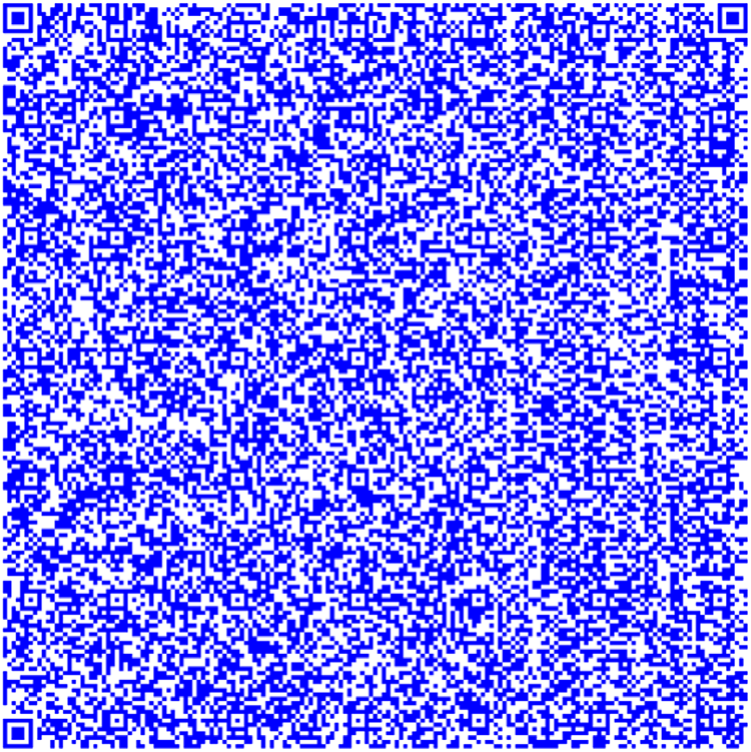
Part 3 of the Chapter One of El Quijote Book encoded in base64.

**Fig. 9:**
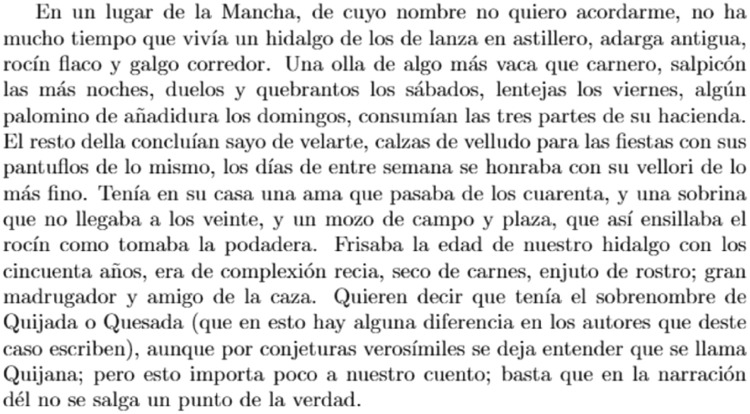
Original text.

**Fig. 10:**
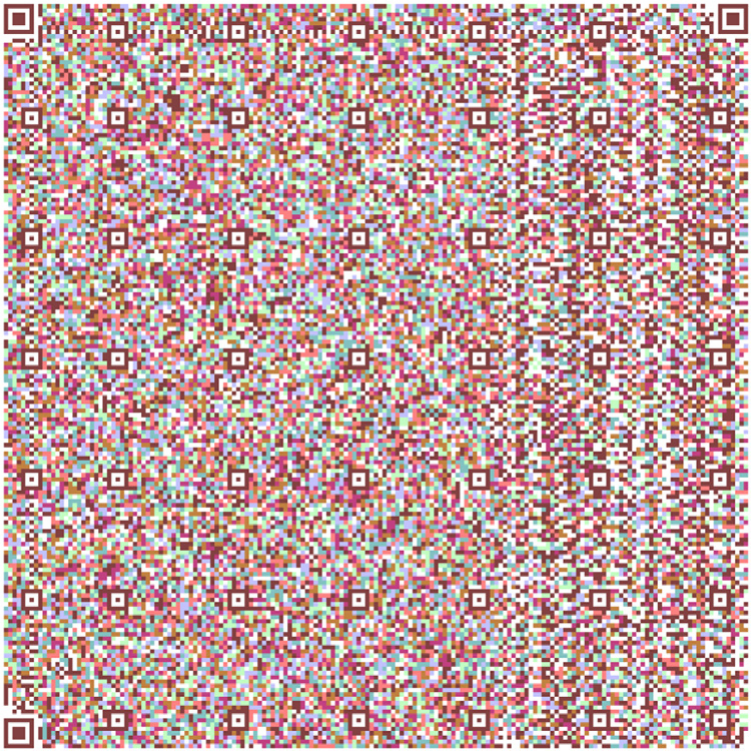
QR code with its text encoded in base64.

In this use case, we will demonstrate an anti-counterfeiting system that displays the encoded information on each page. A function checks this information to prevent external attacks. When the data is converted to base64 code, it is divided into three separate QR codes [18], as seen in Figs. 4, 5, and 6. The QR codes formed are the following:

The QR codes from Figs. 6, 7 and 8 are combined into one QR code. This QR code is connected to a pdf document containing the original content. The following information should be displayed:

This study proposes a novel method to QR code [19] usage by integrating color as a variable that improves both esthetic appeal and practical functionality. The process entails creating QR codes of various colors using a rigorous theoretical framework, which is then validated through practical experience. The results in the Figs. 9 and 10 shows a text with its QR encoded with the text.

**Fig. 11:**
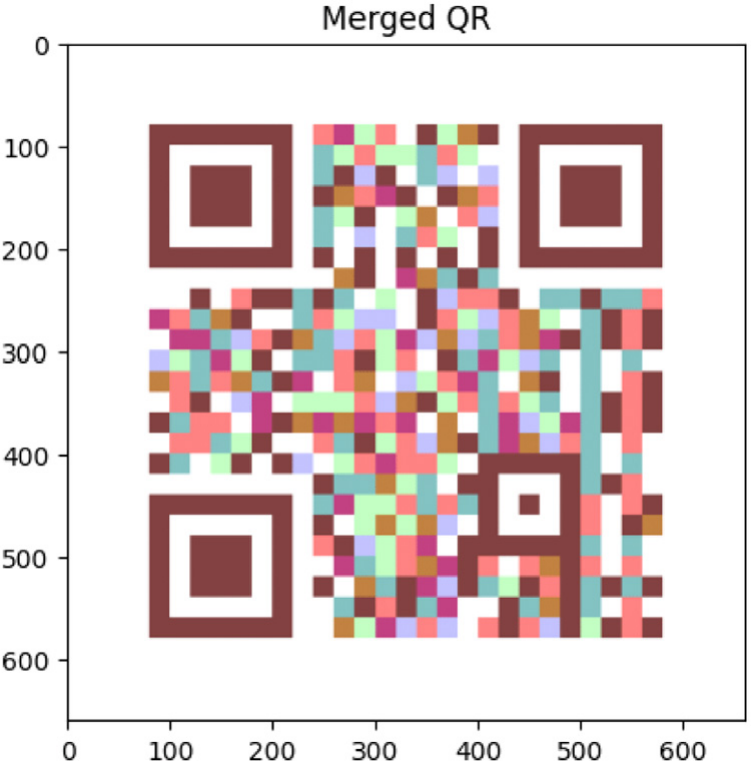
Combine QR Code obtained from the Fig. 1.

In this methodology section we have introduced the literature review, the methodology used and the uses cases. The methodology is based on color codification to code information in one color. The use cases show two cases, one is an example based on the methodology and the second is an app to code text. Then in the method validation we will show the results obtain from the merged QR code and compare it with the theorical. It will be shown the results from the coded text sample and the anti-counterfeiting application. The final part will show the contribution of our research to the scientific world and the future applications.

## Method validation

### Comparing the luminance and the αs between the theorical and practical

Table 1 shows all color mixing values in Equations 4 with the α values from Eqs. (5), (6) and (7). The last column corresponds to the value of the luminance of each pixel obtained from Eq. (9).

**Table 1:** Theorical results obtained from Eq. (9) referred to the Total Luminance with the theorical values of the alphas referred to Eqs. (5), (6) and (7).

Color Code	Red		Green		Blue		Total Luminance (L)
	RGB Coordinate	$\alpha_{r}$	RGB Coordinate	$\alpha_{g}$	RGB Coordinate	$\alpha_{b}$	
Theorical 1	102	0.4	76.5	0.3	76.5	0.3	85
Theorical 2	102	0.4	76.5	0.3	0	0.3	59
Theorical 3	0	0.4	0	0.3	76.5	0.3	25
Theorical 4	0	0.4	0	0.3	0	0.3	0
Theorical 5	0	0.4	76.5	0.3	0	0.3	25
Theorical 6	102	0.4	0	0.3	0	0.3	34

## Results for encoded text and anticounterfeiting system

**Fig. 13:**
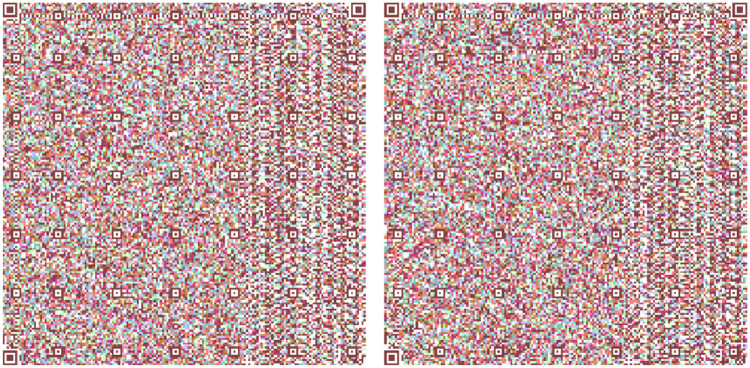
(Left) Model 1 for the use case for encoding the three parts of El Quijote Book in base64 (Right) Model 2 for the use case the use case for encoding the three parts of El Quijote Book in base64 obtained from the Figs. 6, 7 and 8.

Fig. 13 shows the two models used in all the Quijote text to encode the information into base64 which are the results from the combination of Figs. 6, 7 and 8.

This research endeavor has presented an innovative approach to the utilization of QR codes by introducing color as a variable that enhances both esthetic appeal and secure functionality. The color is used as the main variable to code the information and save more information than “standard” QR Code. But our research is focused on coding the main information from others. The aspect from the QR Code can differ from the theorical as it is shown in the Method validation Section.

The methodology involved generating QR codes of different colors through a rigorous theoretical framework, which was further substantiated through practical experimentation. The results have demonstrated the vast array of colors that can be generated by mixing the primary colors, providing insights into potential applications of colored QR codes.

**Fig. 3:**
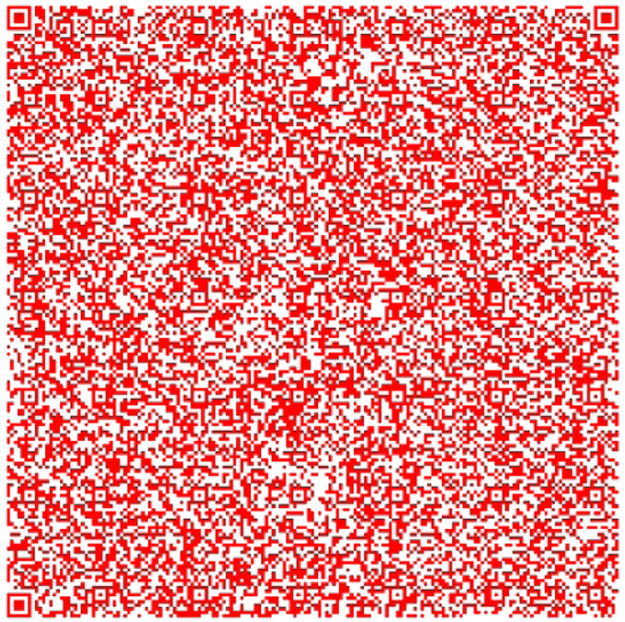
Part 1 of Chapter One of El Quijote Book.

**Fig. 12:**
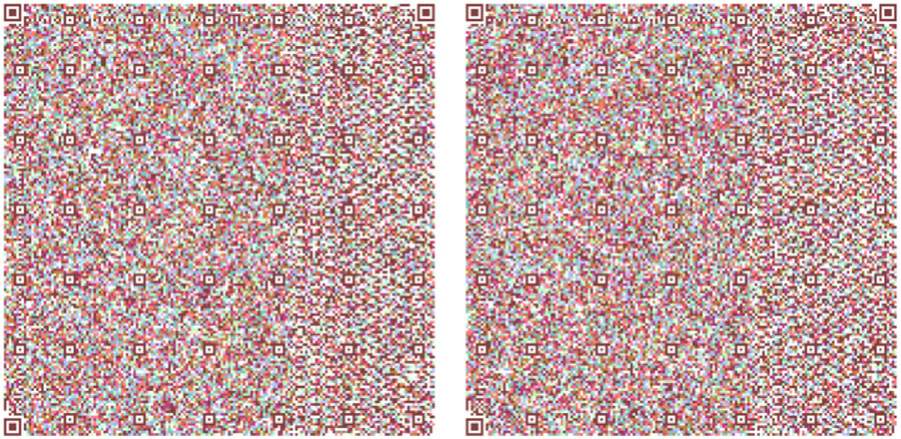
(Left) Model 1 for the use case for encoding the three parts of El Quijote Book (Right) Model 2 for the use case the use case for encoding the three parts of El Quijote Book obtained from the Figs. 3, 4 and 5.

This use case presents an innovative anti-counterfeiting system that incorporates colors as the key to accessing the original text. By encoding the information in Base64 format and distributing it across three distinct QR codes, as demonstrated in Figs. 3, 4, and 5, this system ensures that counterfeiting attempts are significantly thwarted. Furthermore, merging these QR codes into a single entity using a selected α parameter and their inclusion within a PDF document [20,21] alongside the original text adds an extra level of complexity and authenticity verification. This approach not only protects the integrity of the information but also enhances the overall security of the system, making it a robust solution against counterfeiting (Fig. 12,Table 2).

**Table 2:** Results obtained from the combination QR codes from Fig. 2 which are shown in Fig. 11 with the values of Luminance from the Eq. (8).

Red		Green		Blue		Total Luminance (L)	Resulting color
RGB Coordinate	$\alpha_{r}$	RGB Coordinate	$\alpha_{g}$	RGB Coordinate	$\alpha_{b}$		
**255**	**1**	**0**	**0**	**0**	**0**	85	Pure Red
0	0	255	1	0	0	85	Pure Green
0	0	0	0	255	0	85	Pure Blue
255	1	255	1	0	0	170	Yellow
255	1	0	0	255	1	170	Magenta
0	0	255	1	255	1	170	Cyan
255	1	255	1	255	1	255	White
255	1	165	0.65	0	0	140	Orange
255	1	0	0	127	0.50	127.33	Pink
127	0.50	255	1	0	0	127.33	Chartreuse
0	0	255	1	127	0.50	127.33	Spring Green
127	0.50	0	0	255	1	127.33	Violet
0	0	127	0.50	255	1	127.33	Azure
0	0	0	0	0	0	0	Black

Finally, the research has highlighted the potential for color encoded QR codes in various domains, from coding text to create anti-counterfeiting application. This color codification has proven to be a novelty technology which can be used in multiple domains.

## Contribution of the current research

This research has contributed to the scientific world due to the implications of the codification to save the information from external sources. It has been proven that can be combined with the serverless technology [22] to encrypt and decrypt information faster than other systems. This technology is used in the EYE (Economy bY spacE) to code the “sensitive information”.

## Future applications

This research can be improved through quantum colors. These quantum colors can have medium state and create more diverse merged QR colors. It means more variability and more information in the same QR code.

## Ethics statements

This method did not involve studies with living things.

## CRediT authorship contribution statement

**Sara Ignacio-Cerrato:** Conceptualization, Methodology, Software, Investigation, Writing – original draft. **David Pacios:** Conceptualization, Software, Investigation, Writing – original draft. **José Miguel Ezquerro Rodriguez:** Conceptualization, Writing – review & editing, Supervision, Project administration. **José Luis Vázquez-Poletti:** Writing – review & editing, Supervision, Project administration. **Nikolaos Schetakis:** Validation, Writing – review & editing, Supervision, Project administration. **Konstantinos Stavrakakis:** Validation, Writing – review & editing, Supervision, Project administration. **Alessio Di Iorio:** Validation, Writing – review & editing, Supervision, Project administration, Funding acquisition. **María Estefanía Avilés Mariño:** Writing – review & editing, Supervision.

## Declaration of competing interest

The authors declare that they have no known competing financial interests or personal relationships that could have appeared to influence the work reported in this paper.

## Data Availability

The data that has been used is confidential.
